# 

**Published:** 1828-08

**Authors:** 


### MONTHLY LIST OF MEDICAL BOOKS.

[*Medical Works cannot be entered on this List except a copy be sent for the purpose; the titles of Books having frequently been transmitted to us, as published, which have not appeared for weeks, or even months, after.*]

Commentaries on the Causes, Forms, Symptoms, and Treatment, Moral and Medical, of Insanity. By GEORGE MAN BURROWS, M.D. Member of the Royal College of Physicians of London, &c. &c.—8vo. pp. 716. T. and G. Underwood, London, 1828.

A Series of Observations on Strictures of the Urethra, with an Account of a new Method of Treatment. By RICHARD ANTHONY STAFFORD.—8vo. pp. 158. Longman and Co. London, 1828.

A Rational Exposition of the Physical Signs of the Diseases of the Lungs and Pleura. By C. J. B. WILLIAMS, M.D.—8vo. pp. 191. Underwoods.

The Morbid Anatomy of the Bowels, Liver, and Stomach, illustrated by a Series of Plates; with explanatory Letterpress. By JOHN ARMSTRONG, M.D. &c. &c. Fasciculi 1 and 2.—4to. Baldwin and Cradock.

Remarks on the Supply of Water to the Metropolis; with an Account of the Natural History of Water, and of the Chemical Composition and Medical Uses of all the known Mineral Waters. By MICHAEL RYAN, M.D. &c. &c.—Longman and Co. 1828.

